# Two Community Clusters of Legionnaires’ Disease Directly Linked to a Biologic Wastewater Treatment Plant, the Netherlands

**DOI:** 10.3201/eid2410.180906

**Published:** 2018-10

**Authors:** Anna D. Loenenbach, Christian Beulens, Sjoerd M. Euser, Jeroen P.G. van Leuken, Ben Bom, Wim van der Hoek, Ana Maria de Roda Husman, Wilhelmina L.M. Ruijs, Alvin A. Bartels, Ariene Rietveld, Jeroen W. den Boer, Petra S. Brandsema

**Affiliations:** European Centre for Disease Prevention and Control, Stockholm, Sweden (A.D. Loenenbach);; National Institute for Public Health and the Environment, Bilthoven, the Netherlands (A.D. Loenenbach, J.P.G. van Leuken, B. Bom, W. van der Hoek, A.M. de Roda Husman, W.L.M. Ruijs, A.A. Bartels, P.S. Brandsema);; Municipal Health Service Hart voor Brabant, ’s-Hertogenbosch, Tilburg, the Netherlands (C. Beulens, A. Rietveld);; Regional Public Health Laboratory Kennemerland, Haarlem, the Netherlands (S.M. Euser, J.W. den Boer);; Utrecht University, Utrecht, the Netherlands (A.M. de Roda Husman)

**Keywords:** Legionnaires’ disease, Legionella pneumophila, waterborne diseases, environmental exposure, disease outbreaks, Netherlands, bacteria

## Abstract

A biologic wastewater treatment plant was identified as a common source for 2 consecutive Legionnaires’ disease clusters in the Netherlands in 2016 and 2017. Sequence typing and transmission modeling indicated direct and long-distance transmission of *Legionella*, indicating this source type should also be investigated in sporadic Legionnaires’ disease cases.

In autumn 2016, six reported cases of Legionnaires’ disease (LD) were linked to the town of Boxtel, the Netherlands. In the second half of 2017, eight more cases were identified among residents of the town. During 2003–2015, only 1 non–travel-related LD case was reported in Boxtel. In 2016 and 2017, the cases were investigated to determine if they were linked to a common source. We describe the epidemiologic, environmental, and microbiologic investigation of these 2 *Legionella* clusters.

## The Study

**Figure 1:**
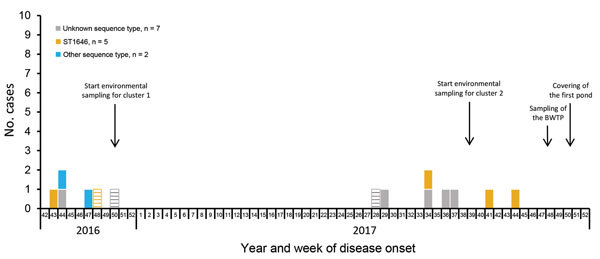
Legionnaires’ disease cases, by sequence type and week of disease onset, Boxtel, the Netherlands, October 2016–December 2017. BWTP, biologic wastewater treatment plant; ST, sequence type.

We defined cases as *Legionella* pneumonia in a person with illness meeting the European Union case definition (*1*) who resided in or visited Boxtel 2–14 days before disease onset during 2016–2017. The 2016 cluster (cluster 1) consisted of 4 residents and 2 nonresidents who work in the industrial area of Boxtel. The onset of disease symptoms ranged from October 28 to December 11, 2016. During July 10–November 3, 2017, seven more cases (all in Boxtel residents) occurred (cluster 2) (Figure 1). Further investigation identified another case, in a person who visited Boxtel 5 days before symptom onset.

The median age of the 14 patients was 72 years (range 51–93 years); 8 patients (57%) were male (Table 1). All 14 patients were hospitalized, 7 (50%) were smokers, and 11 (79%) had co-morbid conditions.

**Table 1:** Characteristics of 14 case-patients in 2 community clusters of Legionnaires’ disease directly linked to a biologic wastewater treatment plant, Boxtel, the Netherlands, October 2016–December 2017*

Characteristic	No. (%)				
	Cluster 1, n = 6	Cluster 2, n = 8		Clusters 1 and 2, N = 14	
Patient characteristics					
Median age >72 y				5 (35.7)	
Sex				8 (57.1)	
M				8 (57.1)	
F				6 (42.9)	
Smoking				7 (50.0)	
Co-morbid conditions				11 (78.6)	
Hospital admission				14 (100)	
ICU admission					
Death				0	
Diagnostic results					
*Legionella* culture performed				6 (42.9)	
LP sg1 culture positive				5 (35.7)	
Urine antigen test positive				14 (100)	
LP PCR positive				4†	
Typing results					
ST1646	2 (33.3)	3 (37.5)		5 (35.7)	
Non-ST1646‡	2 (33.3)	0		2 (14.3)	
No isolate	2 (33.3)	5 (62.5)		7 (50.0)	

*Sources: National Notification Database Osiris; Database National Reference Laboratory for *Legionella*, Haarlem, the Netherlands. ICU, intensive-care unit; LP, *Legionella pneumophila*; ST, sequence type. †Number of PCR tests performed is not available; only number of tests with positive results known.  ‡Obtained through nested PCR with direct sequence–based typing.

Patient interviews did not identify any common exposure, and none of the case-patients had recently traveled abroad. Mapping cases based on the patients’ residential postal code and prevailing wind direction (mainly south-west during individual incubation times [https://www.knmi.nl]) indicated that the source could be within the industrial area of Boxtel.

In November 2016, environmental samples were collected at several potential sources, including a fountain and 5 wet cooling towers (WCT) (Table 2). However, no *Legionella* were detected in these samples. The emergence of new LD cases in 2017 led to the reexamination of these locations, along with identification of additional potential sources, including a biologic wastewater treatment plant (BWTP) in the industrial area. The installation, which was transformed into a BWTP for energy production in summer 2015, consisted of 3 ponds with different degrees of aeration. All ponds tested positive for *L. pneumophila* (Table 2). Because the BWTP effluent drains to the municipal wastewater treatment plant (MWTP), located in the northwest of Boxtel, and discharges onto the Dommel River after treatment, these locations were also sampled. Subsequently, 2 air scrubbers near the BWTP were tested, and air above the BWTP was sampled.

**Table 2:** Results of environmental samples taken during investigation of 2 community clusters of Legionnaires’ disease directly linked to a biologic wastewater treatment plant, Boxtel, the Netherlands, October 2016–December 2017*

Location of samples	Sampling date, wk/y	CFU/L	*Legionella pneumophila* serogroup	ST
Water samples†				
BWTP				
Pond 1 (low aeration)	48/2017	2.0 × 10^6^	sg1	‡
	50/2017	5.6 × 10^7^	sg1	ST1646
Pond 2 (no aeration)	48/2017	7.1 × 10^8^	sg1	ST1646
	50/2017	19.2 × 10^8^	sg1	ST1646
Pond 3 (high aeration)	48/2017	15.0 × 10^8^	sg1	ST1646
	50/2017	22.6 × 10^8^	sg1	‡
Municipal waste water treatment plant				
Influent	39/2017	1.0 × 10^5^	sg1	ST1646
Pond	39/2017	2 × 10^3^	sg1	ST1646
River midstream	39/2017	2 × 10^3^	sg1	ST1646
Riverside	39/2017	2 × 10^4^	sg1	ST1646
Fountain, city center	50/2016	Negative		
	39/2017	Negative		
WCTs and air scrubbers next to BWTP, industrial area				
	50/2016	Negative		
	39/2017	Negative		
	48/2017	Negative		
4 WCTs, industrial area	50/2016	Negative		
5 WCTs, industrial area	39/2017	Negative		
Misting device, industrial area	39/2017	Negative		
Other environmental samples†				
Biologic waste water treatment plant				
Air sample inside pond 3 tent	50/2017	Positive	sg1	‡
Swab inner surface pond 3 tent	50/2017	Positive	sg1	ST1646

*Source: Database National Reference Laboratory for *Legionella*, Haarlem, the Netherlands. BWTP, biologic wastewater treatment plant; sg, serogroup; ST, sequence type; WCT, wet cooling tower. †Water samples were analyzed with an accredited in-house developed method based on ISO 11731:2017, with an additional step of acid treatment. Air samples were collected using a SASS 2300 air sampler (Research International, Seattle, WA, USA), and analyzed in with an accredited in-house developed method based on ISO 11731:2017. ‡Sequence type profile was incomplete because of technical problems experienced during *mompS* typing; however, for all 6 available alleles, results were in accordance with the ST1646 profile (*flaA*, 2; *pile*, 10; *asd*, 14; *Mip*, 10; *mompS*, not available; *proA*, 4; *neuA*, 11).

All 14 cases were confirmed by urine antigen testing (Table 1). Clinical and environmental isolates were genotyped by using sequence-based typing (SBT), as previously described (*2*,*3*), and compared with the European Working Group for *Legionella* Infectious Sequence-Based Typing Database (http://www.hpa-bioinformatics.org.uk/legionella/legionella_sbt/php/sbt_homepage.php). An identical sequence type (ST), ST1646, was found in 5 patients (2 in cluster 1 and 3 in cluster 2) (Figure 1). Two other sequence types were found for 2 patients in cluster 1 (Table 1). SBT of the environmental isolates from the BWTP, the MWTP, and the river also identified ST1646. This sequence type was also detected in isolates from air sampled above the BWTP pond with the most aeration. *Legionella* was not detected in the other sampled locations (Table 2).

**Figure 2:**
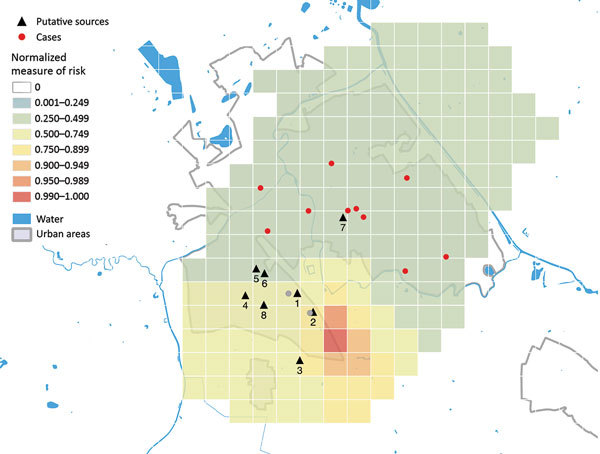
Map of the norrmalized measure of risk for Legionnaires’ disease, Boxtel, the Netherlands, October 2016–December 2017. Results are based on case-patients living in Boxtel who constituted the clusters occurring in 2016 and 2017 (n = 11). Red dots indicate the residential address (postal code) of case-patients. A hotspot is an area with a measure of risk >0.9. Gray dots indicate Legionnaires’ disease cases in nonresidents; these cases are not included in the model. Black triangles indicate the putative sources: 1, biologic waste water treatment plant (industrial area); 2, wet cooling tower next to the biologic wastewater treatment plant (industrial area); 3–6, wet cooling towers (industrial area); 7, fountain (city center); 8, misting device (industrial area).

We used a transmission model for rapid detection of potential environmental sources of airborne pathogens in outbreak investigations (*4*,*5*), which was used for *Legionella* for the first time and applied to the data collected for the outbreak investigation in Boxtel. The model calculated a measure of risk (MR) based on patients’ residential addresses in Boxtel, date of illness onset, and population density. Locations with the highest MR values (hotspots) are likely to contain the actual infection source. The model identified 1 hotspot, located in the southwest of the industrial area, ≈650 m from the BWTP (Figure 2).

To prevent further *Legionella* transmission by aerosols, 2 temporary tents were erected successively to cover the 2 aerated ponds, 1 in December 2017 and the other in January 2018. A permanent solution for covering both aeration ponds is under exploration. After the detection of *Legionella* in the BWTP effluent, a sludge filter defect was identified and repaired. Resampling of the effluent was negative for *Legionella*, indicating that the risk for ongoing contamination of the MWTP and river were reduced.

## Discussion

The LD outbreak in Boxtel occurred in 2 distinct small clusters, rather than a more typical single cluster of cases in a short period. However, the increased LD incidence in the town compared with historical values and the matching sequencing results of clinical isolates suggested a common source for both clusters.

The sequence type ST1646, found in 5 patient isolates and in the environmental samples, identified the BWTP as the most likely source for both LD clusters. Since 2013, this rare sequence type has been detected in 7 other cases in the same region and 2 cases elsewhere in the Netherlands (*3*). ST1646 has not previously been detected in environmental samples (*3*). We were unable to epidemiologically link the other ST1646 cases to Boxtel.

The transmission models outcome, which posited a single hotspot near the BWTP, offers further support for the BWTP as the putative source of infection. The distance of the hotspot, at ≈650 m, is well within the range of a possible source calculated with this model in a previous study (*5*).

Two other clinical strains from cluster 1 were not found in any environmental sample. However, the aeration ponds might have harbored different genotypes. Detection of multiple genotypes causing LD cases from exposure to a single water treatment plant has been previously described (*6*).

BWTPs have been identified as the source of previous LD outbreaks (*6*–*10*). Several risk factors for amplification and transmission of *Legionella* were present in the Boxtel BWTP: a water temperature around 35°C, nutrient-rich water, and aerosol formation through aeration.

Documented outbreaks associated with BWTPs have involved an additional disseminator, such as a WCT or river, in the dissemination of contaminated aerosols, usually marked by a sudden increase in cases. In this outbreak, we assume direct dispersion of bacteria from the BWTP ponds to the patients, which could explain the sporadic nature of the epidemic curve, with 0–2 cases per week spread over 2 periods of 8–16 weeks.

Transmission from WCTs has been described as occurring at a distance of up to 12 km (*11*), whereas direct aerosol dispersal from BWTPs has been detected at a distance of up to 300 m (*8*). In this outbreak, the assumed bacteria transmission from the BWTP ponds to the patients occurred over a distance of >1.6 km. Transmission directly from the elevated aeration ponds is plausible with prevailing wind direction. However, we cannot exclude the possibility that WCTs, air scrubbers, or both in the vicinity of the BWTP disseminated *L. pneumophila*–containing aerosols, although test results for these installations were negative.

Although incidence of community-acquired LD has increased in the Netherlands since 2013 (*12*), infection sources are rarely found (*13*). Because our results indicate direct dispersal over a large distance of >1.6 km, further investigations should consider nontraditional *Legionella* sources, like BWTPs, as possible sources for sporadic LD cases.

The aeration ponds in Boxtel were covered, but whether this measure is sufficient to mitigate all exposure risks involved with this type of installation is still unclear. Because biologic aeration ponds are increasingly used in modern (energy-producing) wastewater treatment installations in the Netherlands, more evaluation is required for the potential health risks associated with BWTPs.
